# The Induced Membrane Technique for the Management of Segmental Tibial Defect or Nonunion: A Systematic Review and Meta-Analysis

**DOI:** 10.1155/2020/5893642

**Published:** 2020-05-22

**Authors:** Chen-An Hsu, Shih-Heng Chen, Soa-Yu Chan, Yi-Hsun Yu

**Affiliations:** ^1^Department of Orthopedic Surgery, Mackay Memorial Hosptial, 10449 Taipei, Taiwan; ^2^Mackay Medical College, New Taipei City 252, Taiwan; ^3^Department of Plastic and Reconstructive Surgery, Chang Gung Memorial Hospital, Chang Gung Medical College and Chang Gung University, 33302 Tao-Yuan, Taiwan; ^4^Department of Computing and Information, Koo Foundation Sun-Yat Sen Cancer Center, Taipei, Taiwan; ^5^Division of Orthopedic Traumatology, Department of Orthopedic Surgery, Musculoskeletal Research Center, Chang Gung Memorial Hospital and Chang Gung University, 33302 Tao-Yuan, Taiwan

## Abstract

**Purpose:**

To identify the predicting factors for union and infection after applying the induced membrane technique (IMT) for segmental tibial defects.

**Methods:**

A systematic review was carried out following the PRISMA guidelines. All databases were searched for articles published between January 2000 and February 2018 using the keywords “Masquelet technique” and “induced membrane technique.” Studies in English reporting more than 5 cases with accessible individual patient data were included. A meta-analysis was performed. Odds ratios (OR) with 95% confidence intervals were calculated.

**Results:**

After reviewing, 11/243 studies (115 patients) were finally selected. The mean age of the patients was 43.6 years (range: 18-84 years), and the mean length of the tibial defect was 5.5 cm (range: 0-20 cm). The multivariate logistic regression analysis revealed that the risk factors of postoperative infection after IMT were infected nonunion (*p* = 0.0160) and defect length ≥ 7 cm (*p* = 0.0291). Patients with postoperative infection after IMT had a lower union rate (*p* = 0.0003). Additionally, the use of an antibiotic polymethyl methacrylate cement spacer reduced the need for surgical revision (*p* = 0.0127). Multiple logistic regression indicated no direct association between the union rate and length of the bone defect.

**Conclusions:**

IMT is a reliable and reproducible treatment for segmental tibial defects. However, initial infected nonunion and defect length greater than 7 cm are risk factors for post-IMT infection, and post-IMT infection was statistically related to nonunion.

## 1. Introduction

Posttraumatic segmental bone defects and recalcitrant nonunions mostly affect the tibia [1, 2]; however, their management is challenging for surgeons. It is generally accepted that bone defects shorter than 6 cm can be treated by nonvascularized autologous bone grafting while the defects longer than 6 cm are managed by other techniques [3, 4] such as distraction osteogenesis [5], free vascularized fibular bone graft [6], allograft [7], titanium cages [8], or even amputation in extreme cases. Among these treatments, distraction osteogenesis and free vascularized bone grafts are among the most common procedures because of their satisfactory results. However, these techniques have some limitations. The treatment course of distraction osteogenesis is long, and the patient may experience inconvenience related to the external fixator, functional disability in daily activities, and pain during bone transportation. Vascularized fibular bone grafts require microsurgical skills, which are technically demanding and not available at every hospital.

Recently, Masquelet introduced a two-stage procedure called the induced membrane technique (IMT) to treat segmental bone defects [9]. During the first stage, after debridement and stabilization of the bone using an external fixator, a polymethyl methacrylate (PMMA) cement spacer is placed in the defect area to maintain limb length, prevent interposed soft tissue between the fracture ends, and induce a biologic membrane. The second stage of the procedure is performed usually 6 to 10 weeks after the first stage and involves careful incision of the induced membrane to remove the spacer. The spacer is later replaced by a large volume of cancellous bone graft harvested from the iliac crest. It is believed that the self-induced membrane serves as a reservoir for bone grafts, which provide osteoinductive growth factors for bone healing.

Since its first description by Masquelet, the IMT has been gradually popularized and used for the treatment of various bone defects. Several modifications of this technique have been described, including fixation with an internal fixator instead of an external fixator during the first stage [10], use of an antibiotic-loaded PMMA spacer [11], and implantation of bone grafts from different sources [12]. Although all reported results of using different modified techniques have been satisfactory, there is no consensus regarding their use. In addition, no review has been dedicated to the treatment of bone defects of the tibia using the IMT. Therefore, the aim of this systematic review and meta-analysis was to identify the factors that contribute to bone union and risk factors for postoperative infection and nonunion after using the IMT for segmental tibial defects.

## 2. Materials and Methods

### 2.1. Search Strategy

**Figure 1:**
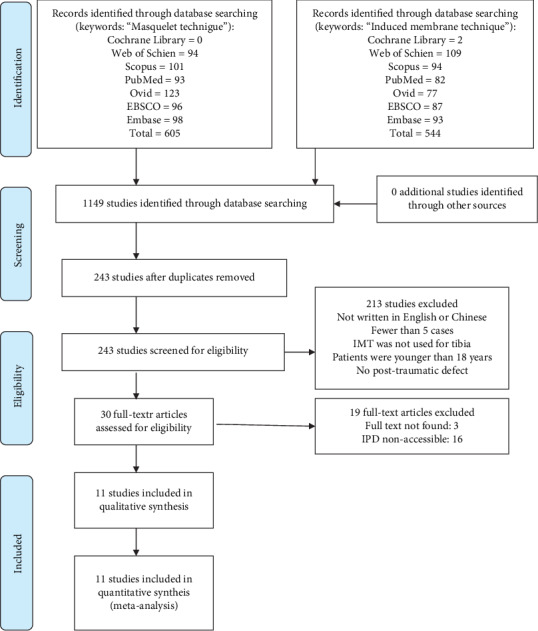
PRISMA flow diagram. IPD: individual patient data.

A systematic review of the medical literature was performed according to the Preferred Reporting Items for Systematic Reviews and Meta-Analyses (PRISMA) statement [13]. A comprehensive search was performed on databases such as the Cochrane Library, Web of Science, Scopus, PubMed, Ovid, EBSCO, and EMBASE, using the keywords “Masquelet technique” and “induced membrane technique.” The time range for our literature search was January 2000–February 2018 (Figure 1). The article selection process comprised two phases: In the first phase, the titles and abstracts were screened for relevance by two independent reviewers after removing the duplicates. In cases of disagreement, a third reviewer was consulted, and uncertain articles were read fully to reach a consensus. During the second phase, full-text articles were obtained and assessed for eligibility.

### 2.2. Eligibility Criteria

Original articles were included if they were written in English, had accessible individual patient data, and reported more than five case descriptions involving patients aged above 18 years with a posttraumatic bony defect or nonunion (either aseptic or septic) of the tibia. Most of the included studies reported cases of various defect locations such as the femur, tibia, fibula, humerus, radius, ulna, and metatarsals. In this study, only cases involving the tibia were extracted from each study for systematic review and further meta-analyses. Data regarding patient demographics (age, sex), nonunion or defect type (infected or non-infected), and defect lengths were obtained. Variations in surgical procedures, such as spacer types (standard or antibiotic), fixation methods (plate, nail, and external fixator), and bone graft sources (iliac crest, reamer-irrigator-aspirator (RIA), nonautologous graft), were recorded. Postoperative infections, additional bone graft surgery, and union status were analyzed to measure clinical outcomes.

### 2.3. Critical Appraisal

Since most studies related to IMT were retrospective case series, the Joanna Briggs Institute Critical Appraisal Checklist for Case Series, comprising 10 items, was used to evaluate the risk of bias [14]. Questions were answered as “yes,” “no,” “unclear,” or “not applicable.”

### 2.4. Statistical Analysis

A meta-analysis was performed using R version 3.5.0. First, we conducted a series of univariate analyses to evaluate the association between patient demographics, treatment-related factors, and clinical outcomes by using the chi-square test and logistic regression; *p* < 0.05 was considered statistically significant. After recognizing several factors that met our preset cut-off for significance, a multivariate analysis was conducted using logistic regression. Results are shown as odds ratios, *p* values, and 95% confidence intervals. Data were illustrated on forest plots to present the effects of significant risk factors.

## 3. Results

A total of 1149 studies were initially identified by searching the database. After removing the duplicates, 243 studies were screened, and 30 studies were included in the second phase to assess their eligibility. Eleven reports [10–12, 15–22] of individual patient data (total of 115 patients) were used for this study. The flow chart of the selection process is presented in Figure 1.

The majority of the included studies were retrospective case series. The risk of bias of each study was evaluated (Table 1). The included patients had a mean age of 43.6 years (range, 18-84 years), and the majority were male (94; 81.7%). All patients underwent the IMT because of a posttraumatic bony defect or nonunion in the tibia. The cases were categorized as infected (66; 57.4%) or noninfected (49; 42.6%). The mean length of the bony defect was 5.5 cm (range, 0 to 15.9 cm) (Table 2).

During the first stage of the procedure, only 22 patients (19%) in two studies received a standard spacer; other patients received an antibiotic-impregnated spacer. An external fixator was most commonly used for fixation during stage one (62; 53.9%) followed by an intramedullary nail (30; 26.1%) and plate (23; 20%). During the second stage of the procedure, fixation was most commonly performed with an intramedullary nail (40; 34.8%), followed by an external fixator (38; 33%) and plate (37; 32.2%). Bone graft sources were the iliac crest (64; 55.7%) or femur (45; 39.1%) which was obtained with an RIA. Nonautologous bone grafts, such as allografts, xenografts, and synthetic and bone substitutes, as well as bone morphogenetic protein (BMP) were also used in some cases (46; 40%). The iliac crest bone graft was more likely used in conjunction with an external fixator (*p* < 0.05) than an RIA during both stages of the IMT and for patients with infected nonunion (*p* < 0.05).

Patient outcomes were evaluated using three main measures: postprocedural infection status after the IMT, repetitive bone graft surgery, and union status. Twenty-eight patients (24.3%) had complications due to infections after the IMT, including pin tract infections and superficial surgical site infections. Eleven patients (9.6%) required further surgeries involving additional bone grafts due to partial consolidation or failure of the bone graft to mature. At the last follow-up examination, 104/115 patients (90.4%) achieved complete union, with healing times ranging from 3 months to 94 months.

A univariate analysis was performed based on individual patient data to determine any possible predictive factors associated with the aforementioned outcome measures. Infection rates after the IMT were higher in cases of initial septic nonunion (*p* < 0.05), in cases involving an antibiotic-free PMMA spacer (*p* < 0.05), and in cases involving intramedullary nail fixation during stage one (*p* < 0.05). Furthermore, using an antibiotic-free spacer increased the need for additional bone graft surgeries (*p* < 0.05). Finally, infected nonunion (*p* < 0.05), the use of an antibiotic-free PMMA spacer (*p* < 0.05), and infection after the IMT (*p* < 0.05) were associated with lower union rates.

**Figure 2:**
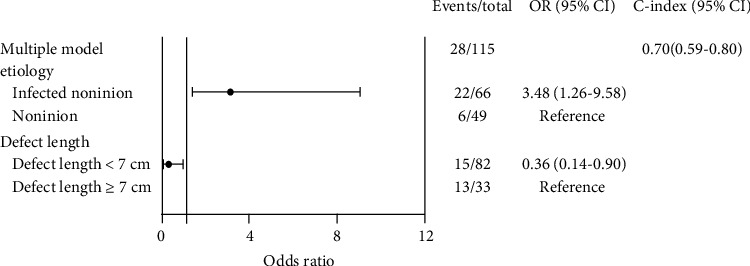
Multivariate analysis of infection complications. The postprocedural infection rate after the IMT was higher for patients with infected nonunion and a defect length ≥ 7 cm.

**Figure 3:**
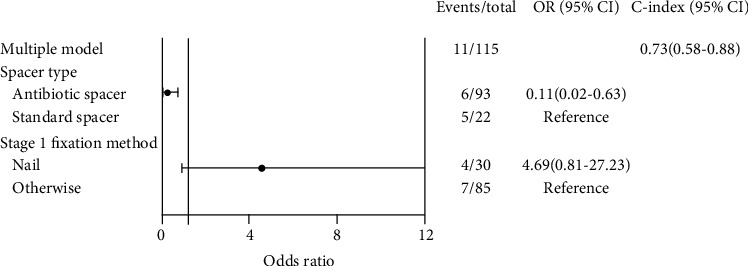
Multivariate analysis of additional bone graft surgery. Use of an antibiotic-impregnated spacer during stage one decreased the risk of regrafting; however, using nail fixation during stage one did not show statistical significance regarding additional bone graft surgery.

**Figure 4:**
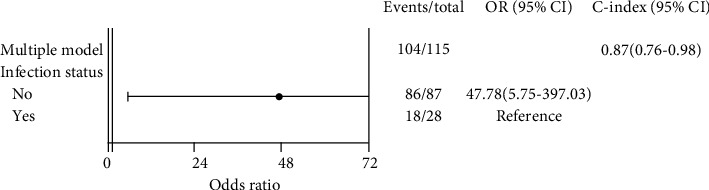
Multivariate analysis of the union status. Patients without postprocedural complications due to infection after the IMT demonstrated better healing outcomes.

**Figure 5:**
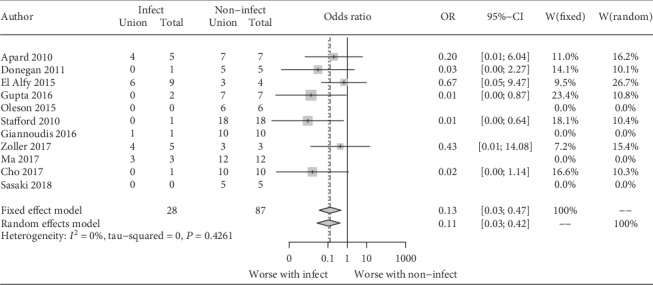
Forest plot of the union status of patients with or without postprocedural complications due to infection after the IMT.

Multivariate logistic regression showed that the predictive factors for infection after the IMT were septic nonunion (*p* < 0.05) and defect length more than 7 cm (*p* < 0.05) (Figure 2). The use of an antibiotic-impregnated spacer during the stage one procedure decreased the need for additional bone graft surgeries (*p* < 0.05) (Figure 3). Patients with infections after the IMT had lower union rates (odds ratio, 0.13; 95% confidence interval, 0.03-0.47); however, there was no direct association between the union rate and defect length (Figure 4). In addition, age, different fixation devices, and different bone graft sources had no significant influence on bone union clinical outcomes. Moreover, there was no significant heterogeneity between the included studies (*I*^2^ = 0%; *p* = 0.4) (Figure 5).

## 4. Discussion

Management of posttraumatic segmental bone defects is challenging for orthopedic and plastic surgeons. Moreover, reconstruction sometimes results in limited functional benefits compared to primary amputations [23]. However, there are several reconstruction techniques for these cases that ensure a better quality of life, including direct autologous bone grafting, distraction osteogenesis [5], and free vascularized fibular bone grafting [6]. The general perception in practice is that these techniques are dependent on the defect size, surrounding soft tissue conditions, available implants, and facility. Essentially, a bone defect larger than 6 cm should be managed with a vascularized bone graft, which is known as the 6 cm rule [24].

The IMT is a relatively new technique [9]. In 2000, a study reported a series of reconstructions of long bone gap nonunion using a two-stage surgery protocol for 35 patients between 1986 and 1999. The IMT is less technically demanding compared to vascularized bone grafting, and its healing time is independent of the defect length compared to bone transport, which requires one-month consolidation period for 1 cm of regenerated bone and distal consolidation between the distal and transported fragments requires 6 months according to Paley et al. [25]. Furthermore, IMT also needs less external fixation time than that required by bone transport because of its two-stage procedure: once the first stage is done without infection complications, external fixator can soon be replaced by plate or nail, providing better life quality for patients. However, at least two invasive surgeries are inevitable with the IMT, and these might result in higher risks of repetitive anesthesia and hospitalization for elderly patients [26]. Grafting at the end of bone transport is still necessary according to Saleh and Rees [27], and data presented by Uzel et al. [28] also reflect the need for bone graft in most scenarios of bone transport technique. Nevertheless, the amount of bone graft needed in bone transport is much smaller than that needed in IMT. Unfortunately, it is hard to compare consolidation time due to missing data in our included studies and in the study presented by Uzel et al. [28]. Both IMT and bone transport are reliable techniques for managing bone defects, and current evidence might not be able to gauge the superiority. Several studies reported the effectiveness of bone union for various bone defect locations and lengths after IMT. To our knowledge, this is the first systematic review to focus on posttraumatic bone defects and nonunion in the tibia treated by the IMT.

We found an association between the bone defect length and infection rates. Because all included patients were initially injured due to trauma, it is possible that larger wounds increased the risk of infection, before and after surgery. However, although the presence of post-IMT infection resulted in significantly lower union rates, no direct relationship between the bone defect length and union rate exists. Although counterintuitive, similar results have been reported previously. Azi et al. performed a meta-analysis to determine the union rate after treatment with the different autologous bone graft techniques and indicated that the bone defect size does not seem to have an impact on bone union [2].

The bone defect length was deemed an important factor in decision-making regarding surgery and in long-term patient outcomes. However, there are too many factors to consider regarding bone union, and substantial evidence of the impact of the defect length is lacking. To reach a more specific conclusion, this study focused on patients with a tibial bone defect who underwent the IMT. In this meta-analysis, defects longer than 7 cm were associated with increased infection rates. However, perioperative infection is multifactorial. Whiting et al. had identified severe soft tissue injury as a risk factor for infection in patients who sustained open tibial shaft fractures treated by intramedullary nailing [29]. Longer bone defect might be associated with more severe compromise of soft tissue, and thereby had higher chances of infection. Because most of the included studies did not clarify the extent of soft tissue defect, further studies should explore these issues in the future. Furthermore, the concept that longer defects may possibly fail seems plausible. The point of view can be supported by El-Alfy, who found that additional surgical procedures were required mainly in patients with big defects (7 cm or more) [15].

Postprocedural complications due to infections after the IMT occurred in 27.3% of patients [1], including surgical site infections and persistence of initial infections. It is considered logical that patients who underwent the IMT due to infected nonunion were much more likely to experience post-IMT infections, and the results of our study revealed the same finding. Postprocedural or recurrent infections after the IMT occurred in 24.3% of patients, including pin tract infections of the external fixator, surgical wound infections, and reactivation of initial infections. Among patients with postprocedural infections after the IMT, 78.6% of them underwent the IMT due to the initial infected nonunion. Because infections could hinder bone healing, it is essential to confirm the eradication of the infection, especially when defects are longer than 7 cm. Therefore, in cases involving suspicious infections even after a successful stage one procedure, we suggest changing the bone cement until the wound heals well and the C-reactive protein level decreases to normal.

Implantation of a PMMA spacer with or without antibiotics is controversial [30]; however, the data analyzed in our study showed that the use of an antibiotic PMMA spacer might prevent repeated surgeries for bone grafting. Among all patients, 9.6% had undergone repetitive bone graft surgeries after the IMT. Two studies have focused on the implantation of antibiotic-free PMMA spacers to prevent concealing infections and bone grafting failure [15, 16]. El-Alfy reported four cases of primary nonunion and graft maturation failure. These cases were later treated by repetitive bone grafting and achieved union [15]. Gupta et al. reported one patient whose bone gap was bridged by the consolidated bone graft but had failed to unite with the host bone at one end. It was later treated with freshening of the bone ends and grafting [16]. Masquelet stated that it is a common mistake to think antibiotic-impregnated spacers are capable of treating bone infections and allow less important debridement [30]. However, our data showed no difference in the union rates of antibiotic-loaded and antibiotic-free PMMA spacers.

Our study had several limitations. First, all included studies were nonrandomized, observational case series with a small number of cases (range, 5-19 cases). Thus, a bias in statistical analysis might exist. However, among the 12 included studies, 5 were processed prospectively. We believe that the prospective data enhances the validity of our analysis. Second, bone union should be based on radiologic evidence such as computed tomography images. However, there was no standard definition of complete union among the included studies. Some studies defined bone union as 3/4 cortices that showed callus formation as a radiological union; however, some used radiographic union score for tibial fractures (RUST) methodology to determine the union status [19, 31]. Furthermore, only few studies provided detailed data regarding comorbidities, bone healing times, and weight-bearing times of individual patient; we tried to contact the authors of each study to obtain this information, but our efforts were unsuccessful. Geographical trends in bone graft selections were evident; for example, RIA bone grafts were used much less often in Asia [20–22]. There was no explanation for this finding, but it might have been related to the different health insurance policies in Asian countries compared to those in European countries or the United States.

## 5. Conclusions

When treating segmental tibial defects, regardless of age, fixation device, and type of bone graft, the induced membrane technique seemed to be a reliable and reproducible solution. Interestingly, the length of bone defect did not significantly decrease the rate of bone union, but was associated with a higher infection rate. However, because of limited individual patient data from the selected articles, there are factors that could not be identified in the meta-analysis, such as whether certain patients healed as infected union were not mentioned. Additional well-designed, randomized, controlled trials are needed to obtain more substantial evidence for these conclusions.

## Figures and Tables

**Table 1:** Risk of bias.

+Low risk of bias - High risk of bias ? Unclear risk of bias X Not applicable	Apard (2010)	Donegan (2011)	El Afly (2015)	Gupta (2016)	Olesen (2015)	Stafford (2010)	Giannoudis (2016)	Zoller (2017)	Ma (2017)	Cho (2017)	Sasaki (2018)
Were there clear inclusion criteria for the case series?	+	+	+	+	+	+	+	+	+	+	+
Was the condition measured in a standard, reliable way for all participants included in the case series?	+	+	+	+	+	+	+	+	+	+	+
Were valid methods used to identify the condition for all participants included in the case series?	+	+	+	+	+	+	+	+	+	+	+
Did the case series include participants consecutively?	-	+	+	+	+	+	+	+	+	+	-
Did the case series involve complete inclusion of participants?	-	+	+	+	+	+	+	+	+	+	-
Was the reporting of the demographics of the participants in the study clear?	+	+	+	+	+	+	+	+	+	+	+
Was the reporting of the clinical information of the participants clear?	+	+	+	+	+	+	+	+	+	+	+
Were the outcomes or follow-up results of cases clearly reported?	+	+	+	+	+	+	+	+	+	+	+
Was the reporting of demographic information of the presenting site(s)/clinic(s) clear?	-	-	-	-	-	-	-	-	-	-	-
Was the statistical analysis appropriate?	X	X	X	X	X	X	X	X	X	X	X

**Table 2:** Patient demographics and preoperative characteristics.

Study	Design	*N* of Cases^1^	Age^2^	Sex^3^	Etiology		Defect length (cm)
					Inf.	N-Inf.	
Apard et al. [10]	Retrospective	12/12	40.7 (18-74)	10/12	7	5	8.67 (5-15)
Donegan et al. [11]	Retrospective	6/11	51 (31-84)	5/6	3	3	6.25 (4-8)
El Afly et al. [15]	Prospective	13/17	43.6 (26-58)	11/13	13	0	7.92 (5-11)
Gupta et al. [16]	Prospective	9/9	35.4 (18-55)	7/9	8	1	5.26 (3.3-8.5)
Olesen et al. [17]	Retrospective	6/8	50.5 (41-70)	5/6	2	4	5.88 (2.9-9.3)
Stafford et al. [12]	Retrospective	19/25	38.7 (23-58)	16/19	5	14	4.76 (1-20)
Giannoudis et al. [18]	Prospective	11/43	39.7 (18-63)	7/11	7	4	4.49 (3.5-7.5)
Zoller et al. [19]	Retrospective	8/9	35.4 (22-53)	6/8	3	5	6 (3-10)
Ma et al. [20]	Retrospective	15/15	53.5 (35-72)	12/15	2	13	0.97 (0-3.5)
Cho et al. [21]	Retrospective	11/21	49.7 (31-74)	10/11	11	0	7.52 (3.4-15.9)
Sasaki et al. [22]	Retrospective	5/7	45.2 (24-77)	5/5	5	0	4.9 (2.5-6)
Total		115/177		94/115	66	49	

Inf.: infected non-union; N-inf.: noninfected nonunion. ^1^Values were given as the number of patients with a tibial defect/total number of patients in each study. ^2^Values were given according to the year as the mean age and age range of patients with only a tibial defect. ^3^Values were given as the number of male patients/total number of patients included in this review.
